# The Proteasome Inhibitor Ixazomib Inhibits the Formation and Growth of Pulmonary and Abdominal Osteosarcoma Metastases in Mice

**DOI:** 10.3390/cancers12051207

**Published:** 2020-05-11

**Authors:** Michael A. Harris, Mark A. Miles, Tanmay M. Shekhar, Carmelo Cerra, Smitha R. Georgy, Stewart D. Ryan, Claire M. Cannon, Christine J. Hawkins

**Affiliations:** 1 Department of Biochemistry and Genetics, La Trobe Institute for Molecular Science, La Trobe University, 3086 Victoria, Australia; M.Harris@latrobe.edu.au (M.A.H.); M.Miles@latrobe.edu.au (M.A.M.); T.Shekhar@latrobe.edu.au (T.M.S.); 18093479@students.latrobe.edu.au (C.C.); 2 Department of Anatomic Pathology, Faculty of Veterinary and Agricultural Sciences, University of Melbourne, 3010 Victoria, Australia; s.georgy@unimelb.edu.au; 3 Translational Research and Animal Clinical Trial Study Group (TRACTS), Faculty of Veterinary and Agricultural Sciences, University of Melbourne, 3010 Melbourne, Australia; stewart.ryan@unimelb.edu.au (S.D.R.); claire.cannon@unimelb.edu.au (C.M.C.)

**Keywords:** osteosarcoma, proteasome inhibitors, bortezomib, ixazomib

## Abstract

Osteosarcoma is the most common form of primary bone cancer. Over 20% of osteosarcoma patients present with pulmonary metastases at diagnosis, and nearly 70% of these patients fail to respond to treatment. Previous work revealed that human and canine osteosarcoma cell lines are extremely sensitive to the therapeutic proteasome inhibitor bortezomib in vitro. However, bortezomib has proven disappointingly ineffective against solid tumors including sarcomas in animal experiments and clinical trials. Poor tumor penetration has been speculated to account for the inconsistency between in vitro and in vivo responses of solid tumors to bortezomib. Here we show that the second-generation proteasome inhibitor ixazomib, which reportedly has enhanced solid tumor penetration compared to bortezomib, is toxic to human and canine osteosarcoma cells in vitro. We used experimental osteosarcoma metastasis models to compare the efficacies of ixazomib and bortezomib against primary tumors and metastases derived from luciferase-expressing KRIB or 143B human osteosarcoma cell lines in athymic mice. Neither proteasome inhibitor reduced the growth of primary intramuscular KRIB tumors, however both drugs inhibited the growth of established pulmonary metastases created via intravenous inoculation with KRIB cells, which were significantly better vascularized than the primary tumors. Only ixazomib slowed metastases from KRIB primary tumors and inhibited the growth of 143B pulmonary and abdominal metastases, significantly enhancing the survival of mice intravenously injected with 143B cells. Taken together, these results suggest ixazomib exerts better single agent activity against osteosarcoma metastases than bortezomib. These data provide hope that incorporation of ixazomib, or other proteasome inhibitors that penetrate efficiently into solid tumors, into current regimens may improve outcomes for patients diagnosed with metastatic osteosarcoma.

## 1. Introduction

Osteosarcoma is the most common form of primary bone cancer and its incidence peaks in adolescence [1]. Osteosarcomas typically arise due to p53 and RB1 mutations in osteoblasts or their precursors in the long bones such as the tibia, femur or proximal humerus [2]. Neoadjuvant administration of chemotherapies including doxorubicin, cisplatin and methotrexate alongside surgery to remove the tumor or affected limb has improved the five-year survival rate for patients diagnosed with osteosarcoma from approximately 20% in the 1960s to 60% in the 1980s [3,4]. Between 20% and 30% of patients diagnosed with osteosarcoma present with pulmonary metastases, and for these patients the five-year survival rate diminishes to only 20% to 30% [5]. Given the lack of improvement in patient outcomes since the 1980s, particularly for those with pulmonary metastases, new therapies are required for osteosarcoma. 

The proteasome is one of many molecular targets identified for the development of novel anti-cancer therapies. The first-generation therapeutic proteasome inhibitor, bortezomib, is FDA-approved for the treatment of multiple myeloma and mantle cell lymphoma [6,7]. Bortezomib induces cell death through proteotoxic stress and altering the equilibrium of pro/anti-apoptotic proteins by inhibiting the degradation of ubiquitinated proteins by the 20S proteasome [8]. Multiple studies have documented the sensitivity of osteosarcoma cells to bortezomib in vitro and in vivo [9,10,11]. Despite promising preclinical data, a phase 1 clinical trial investigating the efficacy of bortezomib in pediatric patients with solid tumors (including two osteosarcoma patients) [12] and a phase II clinical trial investigating the efficacy of bortezomib for treatment of metastatic sarcomas (including a single osteosarcoma participant) [13] concluded that the drug had minimal activity in these contexts as a single agent. Additional studies have shown that bortezomib has poor anti-cancer activity against a range of solid tumors in vivo [14,15]. A review of 32 clinical trials that tested the efficacy of bortezomib against solid carcinomas found no evidence of a therapeutic effect when used as a single agent or when combined with chemotherapies [16]. 

Numerous hypotheses have been postulated to explain the poor responses of solid tumors to proteasome inhibitors [8,17]. For example, TP53 mutations, which are common in solid cancers but rare in multiple myeloma and other hematological cancers [18], decreased sensitivity to bortezomib [19,20]. KRAS mutations, which occur in the majority of pancreatic cancers [21], were also linked to resistance to proteasome inhibition [22]. Discrepancies between the promising in vitro potency of bortezomib towards cells derived from some solid cancer types, versus its minimal efficacy in patients with those solid tumors may be explained by its inability to penetrate tumors. Bortezomib only effectively reduced the growth of well vascularized tumors [23,24,25,26,27]. Researchers have explored strategies to improve the bioavailability of bortezomib: packaging the drug into liposomes that permeate preferentially through tumor vasculature was far more effective at treating xenograft solid tumors than unpackaged bortezomib [28]. 

Second-generation proteasome inhibitors have subsequently been developed including carfilzomib and ixazomib, both of which are FDA-approved to treat relapsed multiple myeloma [29,30]. Ixazomib is the first orally available proteasome inhibitor that exhibited markedly improved activity against solid tumors, relative to bortezomib [15]. In pre-clinical models, ixazomib reduced the growth of solid tumors and enhanced survival compared to bortezomib [15]. Osteosarcoma cells were found to be sensitive to ixazomib in vitro [9,31], but no studies have investigated the anti-osteosarcoma efficacy of ixazomib in vivo. 

One of the challenges in overcoming the stagnant response rates of osteosarcoma patients is that the extremely low incidence of the disease [32] makes it difficult to establish clinical trials to evaluate new therapies. Several of the clinical trials that have occurred in more recent years failed to identify any new treatments that would improve patient outcome over the currently used therapies [13,33,34,35]. Dogs with spontaneous osteosarcoma are a valuable translational model to evaluate new therapies from pre-clinical in vitro and rodent models and improve the likelihood of new treatments being successfully implemented into human patients with osteosarcoma. [36,37]. Dogs and humans with osteosarcoma share similar genetics, tumor-immune interactions, cellular environment, tumor cell morphology, prognostic factors and natural disease course (with a high rate of pulmonary metastasis) and undergo similar treatments including surgery and chemotherapy. A key difference between the two is that the incidence of osteosarcoma in dogs is 27 times higher than humans, facilitating clinical trials [38].

Here we compared the effectiveness of bortezomib and ixazomib in vivo, in mice implanted with intramuscular osteosarcomas or bearing pulmonary and abdominal metastases. Neither proteasome inhibitor was active against the primary tumors, but ixazomib reduced the formation and slowed the progression of pulmonary and abdominal metastases, as well as enhancing survival. Both proteasome inhibitors were more effective against lung tumors that were relatively well vascularized compared to less vascularized intramuscular tumors.

## 2. Results

### 2.1. Established Human Osteosarcoma Cell Lines and Freshly Resected Canine Osteosarcoma Cells Are Sensitive to Proteasome Inhibitors at Physiologically Relevant Concentrations

We previously reported the in vitro sensitivity of canine and human osteosarcoma cells to a panel of proteasome inhibitors including bortezomib and ixazomib [9]. In this study we compared the efficacy of bortezomib to ixazomib in vivo, using human osteosarcoma cell lines that are tumorigenic and metastasize to the lungs of athymic mice [39,40,41]. We first assayed the in vitro sensitivity of these cell lines, and cells isolated from resected canine osteosarcomas.

**Figure 1:**
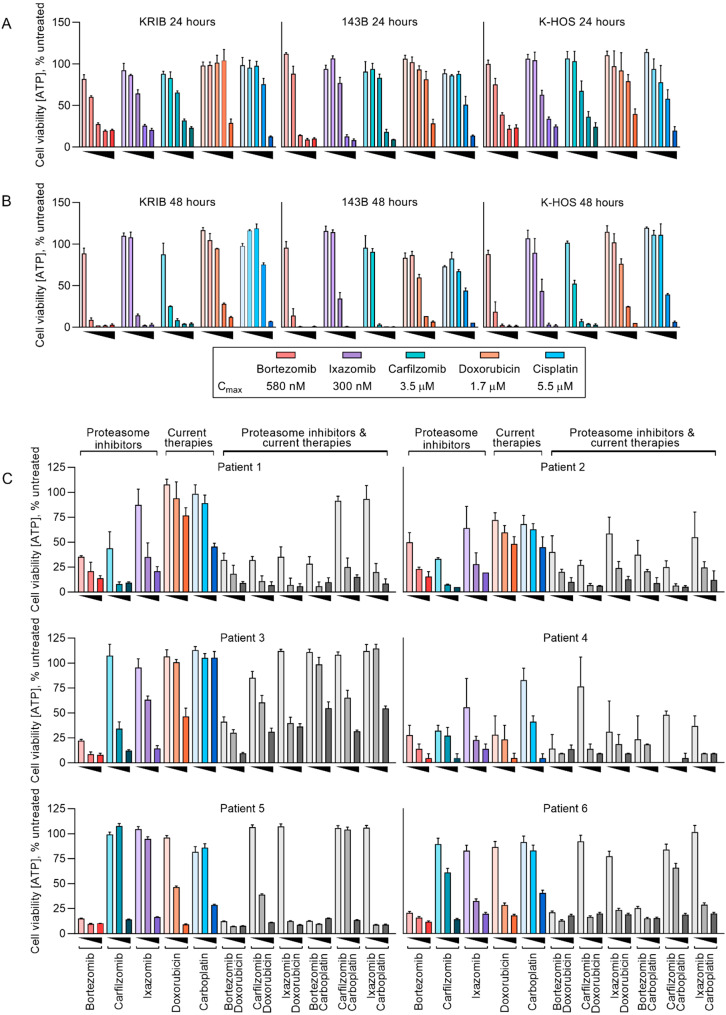
Established and minimally passaged primary osteosarcoma cell lines are more sensitive to proteasome inhibitors than current treatments for osteosarcoma. Established human osteosarcoma cell lines were treated with therapeutic proteasome inhibitors; bortezomib, carfilzomib and ixazomib or current treatments at 0.1%, 1%, 10%, 100% or 10x each drug’s C_max_ for 24 h (**A**) or 48 h (**B**). Cells from six resected canine osteosarcoma tumors were treated with proteasome inhibitors, current treatments for canine osteosarcoma or a combination of each drug class at 1%, 10% or 100% C_max_ for 48 h (**C**). All viabilities reported are relative to untreated samples (n = 3 +/− SEM).

Cells were treated at concentrations between 0.1% and 10x the peak plasma concentration (C_max_) of bortezomib (C_max_ = 580 nM [42]), ixazomib (C_max_ = 300 nM [43]), carfilzomib (C_max_ = 3.5 µM [44]), doxorubicin (C_max_ = 1.7 µM [45]) or cisplatin (C_max_ = 5.5 µM [46]) for 24 (Figure 1A) or 48 h (Figure 1B). After 24 h, the proteasome inhibitors reduced cell viability below 20% in all three cell lines at concentrations below C_max_. In contrast, doxorubicin and cisplatin failed to reduce cell viability below 50% at concentrations equal to or below C_max_. After 48 h, the proteasome inhibitors greatly reduced cell viability at concentrations between 1% and 10% C_max_ whereas doxorubicin and cisplatin only reduced cell viability below 20% at 100% C_max_. Of the three proteasome inhibitors used, bortezomib was the only drug that exerted a robust effect in all cell lines when applied at 1% C_max_. Ixazomib was the least effective, even considering that the molar concentrations of ixazomib used were around half those of bortezomib. This probably reflects the six-fold faster dissociation of ixazomib than bortezomib from the proteasome [15].

While the sensitivity of osteosarcoma cell lines to proteasome inhibitors was promising, we wanted to confirm whether this could be an artifact of in vitro culturing. Canine and human osteosarcomas are extremely similar and share the same treatments of doxorubicin and platinating agents [36,47,48], but osteosarcoma is more common in dogs than people [38], and canine tumors are typically resected without administration of neo-adjuvant chemotherapy that human patients usually receive [49]. These factors led us to assess the ex vivo sensitivity of canine osteosarcoma cells to proteasome inhibitors and chemotherapy drugs. We assayed the sensitivity of cells from six canine osteosarcomas to 48 h exposures to bortezomib, ixazomib, carfilzomib, doxorubicin and carboplatin, the platinating agent typically used to treat dogs: [50] (C_max_ = 224 µM [51]) (Figure 1C). We also combined the proteasome inhibitors with the chemotherapies to determine cooperation between the drugs. Like the established human osteosarcoma cell lines, all six canine samples were sensitive to the proteasome inhibitors, however the chemotherapies were far less effective with the best responses reducing viability by half at C_max_. When the proteasome inhibitors were combined with doxorubicin or carboplatin, the treatments were barely additive. This may be a consequence of doxorubicin’s cytotoxicity being dependent on cell division [52] while proteasome inhibitors halt the cell cycle [9,10].

### 2.2. Ixazomib and Bortezomib Inhibit the Formation and Growth of Pulmonary KRIB Osteosarcoma Metastases But Not Primary Intramuscular Tumors

While the tolerability of 1 mg/kg bortezomib twice weekly is well established [10,15,53], the maximum tolerated dose of ixazomib is less defined in immunocompromised mice: in one study, 5 mg/kg [54] was intolerable, but in another 11 mg/kg biweekly resulted in no weight loss [55]. We found that athymic BALB/c nude mice tolerated 5 mg/kg ixazomib twice weekly for four weeks, but not 7 mg/kg (Supplementary Figure S1).

**Figure 2:**
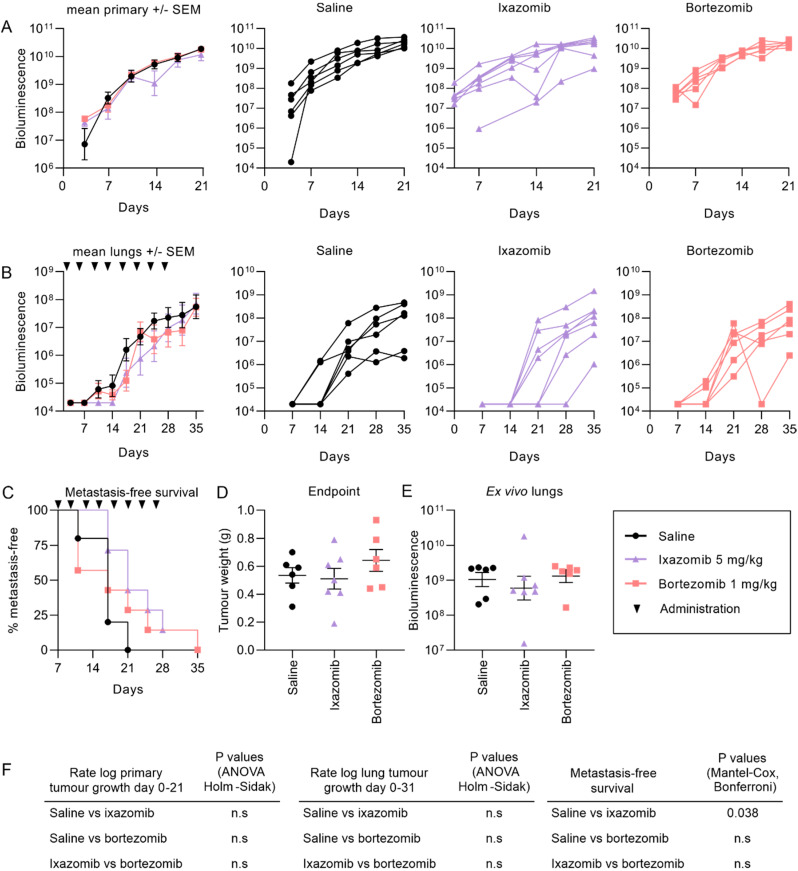
Ixazomib delays the formation of pulmonary metastases in mice bearing intramuscular KRIB osteosarcoma tumors. KRIB-luc cells were implanted into the tibialis anterior muscle of nude mice and one week later commenced treatment with saline, ixazomib or bortezomib, twice weekly for 4 weeks (**A**). Primary tumors were shielded from the camera and mice were imaged facing up twice a week to monitor the growth of lung metastases (**B**). A Kaplan Meier curve was used to compare the rate of formation of detectable metastases in mice between the three treatment groups (**C**). Tumors were removed from mice at the end of the experiment and weighed (**D**). Lungs were removed from the mouse at the end of the experiment and lung tumors imaged by bioluminescence ex vivo (**E**). Rate of growth of tumors between treatment groups and the duration of metastasis-free survival were compared (**F**). (n = 6 for bortezomib and saline and 7 for ixazomib, +/− SEM).

We compared the efficacy of ixazomib and bortezomib in mice bearing intramuscular KRIB-luc tumors, which reliably form lung metastases in two to three weeks [39] to determine if either drug could reduce the growth of primary tumors or inhibit the formation of metastases. The mean bioluminescence readings of the primary tumors in mice treated with saline, bortezomib or ixazomib were similar in the first 21 days of the experiment (Figure 2A) after which the bioluminescent values became unreliable, presumably due to poor uptake of luciferin in larger tumors. By 10 days after cell inoculation, mice began to develop lung metastases, which could be detected by in vivo bioluminescence imaging. The mean growth of lung tumors was lower on average in mice treated with ixazomib or bortezomib than vehicle during the four weeks of treatment, however these differences were not statistically significant (Figure 2B). Promisingly, ixazomib significantly delayed the formation of detectable lung metastases compared to the saline treated mice (Figure 2C), consistent with it having improved pharmacokinetics relative to bortezomib [15]. Primary tumor weights at the endpoint of the experiment were no different between the three treatments (Figure 2D), which was surprising given that ixazomib has been demonstrated to have enhanced solid tumor penetration compared to bortezomib [15]. On average the overall tumor burden in the lungs of ixazomib-treated mice was lower than those that received saline or bortezomib (Figure 2E), however this difference was not significant.

**Figure 3:**
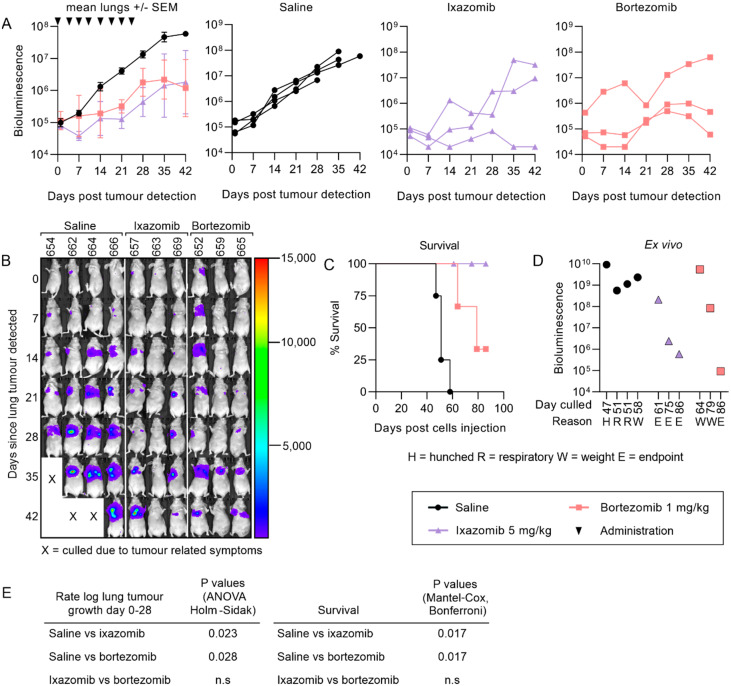
Ixazomib and bortezomib inhibit the growth of KRIB-luc pulmonary metastases and survival is enhanced in ixazomib treated mice compared to mice treated with saline. Mice were injected with KRIB-luc cells intravenously and alternately allocated to different treatment groups as lung tumors were detected and subsequently treated for four weeks (**A**). Compiled images of bioluminescence representing tumor growth starting from the day the tumor was detected until the endpoint of the experiment (**B)**. A Kaplan Meier curve was used to compare survival times between treatment groups (**C**). Lungs were removed from the mice when tumor related symptoms required the mouse to be euthanized or at the endpoint of the experiment and imaged ex vivo to compare endpoint tumor burden in the lungs (**D**). Rates of growth of tumors and survival between treatment groups were compared (**E**). (n = 4 for saline and 3 for ixazomib and bortezomib, +/− SEM).

The presence of treatment-resistant primary tumors, which could continuously seed cells via the circulation to the lungs, may have confounded evaluation of the efficacy of the proteasome inhibitors against lung metastases. To address this, we explored the anti-metastatic efficacy of ixazomib compared to bortezomib or saline further in mice bearing “experimental” lung metastases in the absence of primary tumors. This was achieved by intravenous injection of KRIB-luc cells, which we previously determined could form lung metastases [41]. Mice were imaged twice weekly and allocated alternately to a treatment group the day after bioluminescence was detected in their lungs. Two ixazomib treated mice and one bortezomib treated mouse that had undetectable or small metastases with stable growth two weeks after their final treatment were kept alive until 70 days post tumor cell detection. All other mice were culled two weeks after their final treatment. Both ixazomib and bortezomib slowed the growth of established lung metastases compared to mice receiving saline (Figure 3A,B). Ixazomib also enhanced the survival of mice (Figure 3C) and resulted in a lower tumor burden in the lungs when measured ex vivo (Figure 3D) at the endpoint of the experiment (despite surviving longer than saline- or bortezomib-treated mice). While all saline-treated mice and two bortezomib-treated mice required euthanasia due to intolerable tumor-related symptoms that developed after the final treatments, all ixazomib treated mice remained asymptomatic throughout the experiment (Figure 3C).

**Figure 4:**
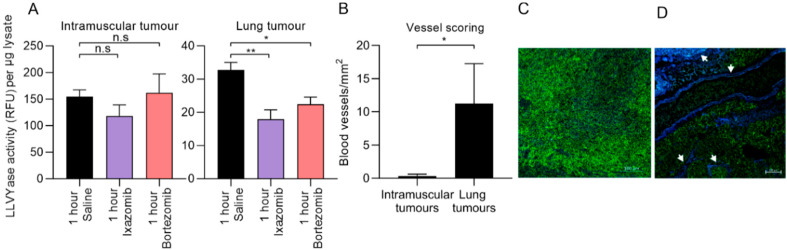
Ixazomib and bortezomib inhibit the proteasome activity of vascularized lung tumors but not poorly vascularized intramuscular tumors. Tumor tissue was resected one hour after mice were treated with either saline, ixazomib or bortezomib and assayed for LLVYase activity (**A**). Sections of primary tumor and lung tissue containing metastases were stained for CD44 (green) and CD31 (blue) positive cells to quantify blood vessels (**B).** Arrows indicate blood vessels in representative pictures of stained primary tumor (**C**) and lung tumors (**D**). (**A**) One-way ANOVA with Holm-Sidak corrections were used to determine the likelihood that differences in LLVYase activity in treated and untreated tissue samples were due to random chance. (**B**) An unpaired t-test was used to determine if the differences in blood vessel counts between tissue types were due to random chance (n.s *p* > 0.05, * *p* < 0.05, ** *p* < 0.01; n = 2 biological replicates for LLVYase activity and 3 biological replicates for blood vessel scoring +/− SEM). (**C**,**D**) Scale bars represent 100 µm.

Other studies have reported a relationship between the effectiveness of bortezomib against solid tumors and their vascularization [27]. We assayed for LLVYase activity, which reflects chymotrypsin-like activity of the proteasome [56] in tumors from treated and untreated mice. We chose to assay the chymotrypsin-like protease activity because bortezomib and ixazomib inhibit this proteolytic activity with similar low nanomolar potencies, and both drugs inhibit it around 10-fold more efficiently than proteasomal caspase-like activity and around 1000-fold more efficiently than the trypsin-like activity [15]. We therefore estimate that this assay detects around 90% of the total proteolytic activity of the proteasome, and because the drugs have very similar specificities, the assay probably provides a fair comparison of their inhibitory actions in these tissues. Both proteasome inhibitors significantly inhibited LLVYase activity in lung tumor tissue compared to saline, whereas neither drug inhibited LLVYase activity in intramuscular tumor tissue compared to saline (Figure 4A). Vascularity in primary intramuscular tumors and lung metastases was quantified by immunofluorescence, which revealed an increased density of CD31 positive blood vessels in CD44 positive lung metastases compared to primary tumors (Figure 4B–D).

### 2.3. Ixazomib Inhibits the Growth of 143B Osteosarcoma Metastases and Enhances Survival Compared to Saline Treated Mice

**Figure 5:**
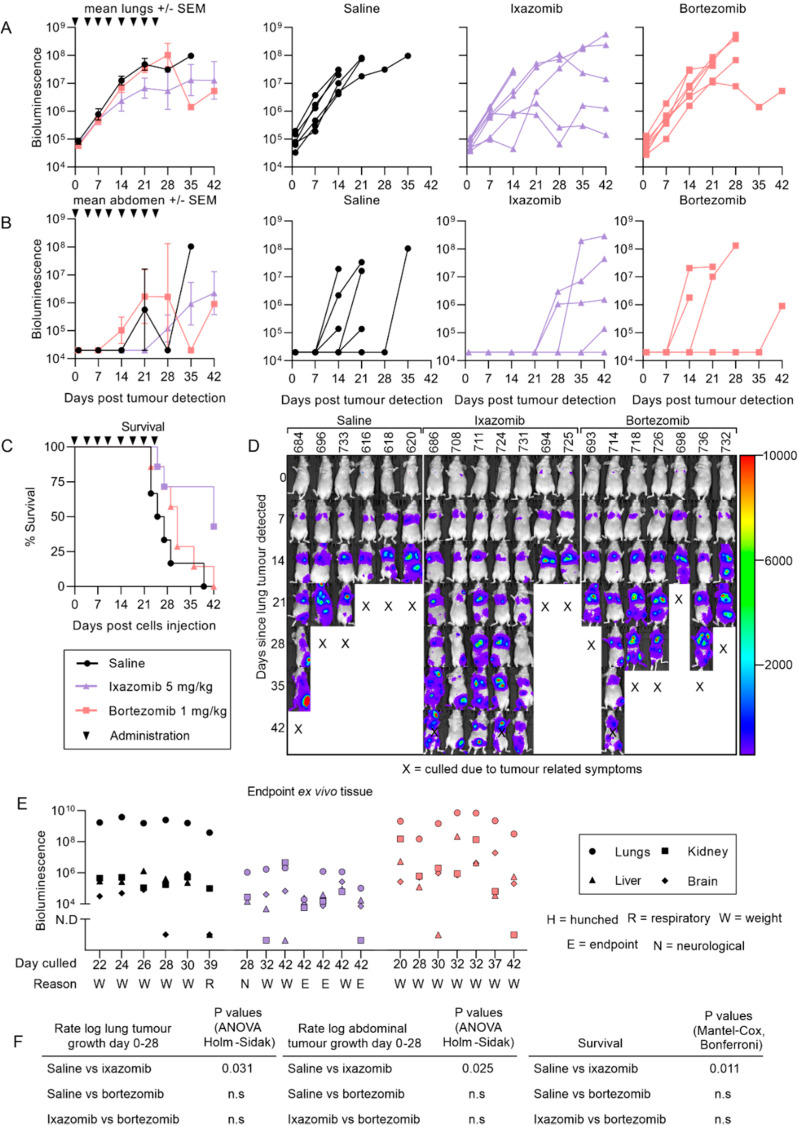
Ixazomib reduces the growth of pulmonary and abdominal metastases and enhances the survival of mice bearing 143B-luc tumors. Mice were injected with 143B-luc cells intravenously, ranked based on their lung bioluminescence when this was detected (which was three or seven days later) and alternately distributed among treatment groups. Mice were imaged once per week thereafter, to monitor pulmonary (**A**) and abdominal (**B**) metastases. A Kaplan Meier curve was used to compare survival time between treatment groups (**C**). Compiled images of bioluminescence representing tumor growth starting from the day the tumor was detected until the endpoint of the experiment (**D**). When tumor related symptoms required the mouse to be euthanized or at the endpoint of the experiment, lungs, liver, kidney and brains were removed from mice and imaged for tumors by bioluminescence ex vivo to compare overall tumor burden in each mouse between treatment groups (**E**). Rates of growth of tumors and survival between treatment groups were compared (**F**). (n = 6 for saline and 7 for ixazomib and bortezomib, +/− SEM).

Osteosarcoma has also been reported to metastasize to organs other than the lungs in some cases [57,58]. We have previously described an aggressive osteosarcoma model where luciferase-tagged 143B cells injected intravenously into nude mice formed lung, kidney and liver metastases in less than two weeks [41]. Unlike the KRIB metastatic model, only ixazomib reduced the growth of 143B lung tumors whereas bortezomib was ineffective (Figure 5A). Ixazomib, not bortezomib, also delayed the formation of abdominal metastases (liver and/or kidneys) compared to saline (Figure 5B). Ixazomib-treated mice survived longer and some were asymptomatic at the endpoint of the experiment, whereas most saline- and bortezomib-treated mice required euthanasia due to intolerable tumor-related symptoms (Figure 5C–E). The most striking difference between ixazomib, compared to saline and bortezomib, was the reduced overall tumor burden in the lungs, liver and kidneys ex vivo (Figure 5E). The ex vivo bioluminescence of the lungs in ixazomib-treated mice was at least 100-fold lower than the mice treated with saline or bortezomib, despite being culled up to 21 days later.

### 2.4. Resected KRIB-luc and 143B-luc Osteosarcoma Cells Do Not Acquire Resistance During In Vivo Treatment with Proteasome Inhibitors

**Figure 6:**
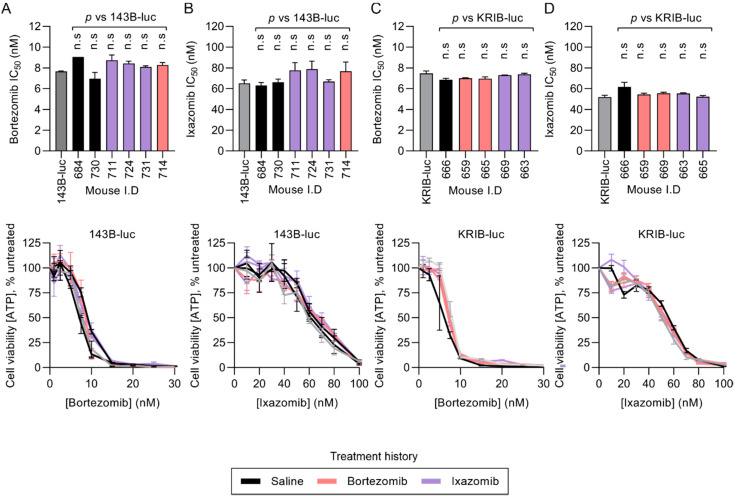
Cells from resected KRIB-luc and 143B-luc lung tumors are as sensitive to proteasome inhibitors as parental cells that have not been implanted in mice, regardless of in vivo treatment history. 143B-luc (**A**,**B**) and KRIB-luc (**C**,**D**) lung tumors were resected from mice following 4 weeks of treatment with saline, ixazomib or bortezomib. Cells isolated from resected tumors (black and colored columns), and in vitro-cultured cells (gray columns), were exposed in vitro to a range of concentrations of bortezomib (**A**,**C**) or ixazomib (**B**,**D**) and residual ATP was measured to assess sensitivity. Two-way ANOVAs with Bonferroni corrections were used to determine the probability that differences in the sensitivity of cells from resected tumors compared to naïve parental cells were due to random chance (n.s *p* > 0.05); n = 3 technical replicates +/− SEM).

To determine if osteosarcoma cells acquired resistance during in vivo treatment with either bortezomib or ixazomib, we resected and disaggregated 143B-luc and KRIB-luc lung metastases for ex vivo sensitivity analysis. In vivo exposure to proteasome inhibitors (or saline) did not affect the in vitro sensitivity of 143B-luc cells (Figure 6A,B) or KRIB-luc cells (Figure 6C,D) to bortezomib or ixazomib. The similar sensitives of the ex vivo treated cells compared to naïve parental cells to the proteasome inhibitors suggests that any poor efficacy observed in vivo may relate to the local concentration of the drug experienced by the osteosarcoma cells in vivo.

## 3. Discussion

The stagnant progression in the outcome of patients diagnosed with pulmonary osteosarcoma metastases since the 1980s [4] underpins the urgent need for therapies to be developed or repurposed to treat metastatic osteosarcoma. Several studies have reported the toxicity of bortezomib to osteosarcoma cells [9,10,11,59], however none of the osteosarcoma patients who have received bortezomib in clinical trials exhibited objective responses [12,13]. The second-generation proteasome inhibitor ixazomib, which was shown to possess enhanced solid tumor penetration compared to bortezomib [15], was reported to also be toxic to osteosarcoma cells in vitro [9,31] but its potential had not been evaluated in vivo. In this study, we compared the efficacy of ixazomib to bortezomib in pre-clinical models of primary and metastatic osteosarcoma in nude mice. While both drugs inhibited the growth of KRIB-luc lung tumors, only ixazomib reduced the growth and formation of 143B-luc metastases and prolonged survival in both metastatic models.

The in vitro response of established human cell lines and primary canine osteosarcoma cells to proteasome inhibitors was extremely homogenous: all three proteasome inhibitors substantially reduced cell viability at their respective C_max_. In contrast, the chemotherapies only appeared to be effective at concentrations equal to or exceeding their peak concentration achievable in patients. The substantial in vitro sensitivity to proteasome inhibitors implies that, unlike other solid cancer types in which resistance to proteasome inhibition may reflect intrinsic resistance [8,17], osteosarcomas would be expected to respond to proteasome inhibitors if intratumoral levels could be achieved that resemble those in the blood.

Ixazomib was able to inhibit the growth of the pulmonary metastases in both models of metastatic osteosarcoma used in this study, but all mice had detectable lung tumors when examined ex vivo at the endpoint of the experiments. Unfortunately, the nude mice used in this study were more sensitive to ixazomib-induced toxicities than previously reported [15]: they failed to tolerate administration exceeding 5 mg/kg twice per week. It is possible that increasing the frequency in administration or dose of ixazomib would elicit greater anti-tumor activity than we observed if intolerable symptoms could be avoided, but it is not clear how the therapeutic window in different mouse strains would compare to that in human (or canine) patients. Alternatively, the sub-curative efficacy of ixazomib against metastatic osteosarcoma may augment responses to current osteosarcoma treatments. Several pre-clinical studies and clinical trials have already highlighted cooperation or synergy when bortezomib or ixazomib are combined with platinating agents [60,61,62,63,64] or doxorubicin [65,66] to treat solid tumors. The cooperation between these drug classes may be a result of proteasome inhibitors improving the uptake of platinating agents into cells [67] or enhancing the ability of chemotherapies like doxorubicin to penetrate solid tumors by reducing the density of the extracellular matrix [65]. 

Given the reportedly enhanced solid tumor penetration of ixazomib over bortezomib [15], we were disappointed that neither was effective against primary intramuscular tumors. Consistent with this observation, lysates from primary tumors resected from treated and untreated mice harbored similar proteasome activity. Those data reflect the average LLVYase activities one hour after drug administration. Peripheral regions of treated tumors, or areas surrounding the very rare vessels, may have experienced drug-mediated suppression of proteasome activity at this time, which was not evident in assays of total lysates. A time course analysis may have revealed slower drug penetration into areas more distant from vessels. In contrast, equivalent analysis of lung tumors, which were better vascularized, did reveal a difference in average proteasome activity between tumors from treated and untreated mice, which aligned with our observation that the drugs had greater impact on the growth of metastases than primary tumors. Thus, vascularity may account for the differing responses of intramuscular versus lung tumors to treatment. Similar results have also been published in a study comparing the effectiveness of bortezomib against well- and poorly-vascularized solid tumors [27]. Our work extends these findings and suggests that this is likely a feature of proteasome inhibitors as a class. Intriguingly, we detected lower proteasome activity in untreated lung tumors than untreated primary tumors, suggesting a biochemical adaptation to the different microenvironments (lung versus muscle). Maybe the lower basal proteasome activity in lung tumors rendered those cells more sensitive to the toxic effects of proteasome inhibition. Further work will be needed to explore these possibilities. The limited efficacy of bortezomib and ixazomib against the primary tumors could also be a consequence of the anti-angiogenic properties of proteasome inhibitors in general [68], which could suppress the efficacy of chemotherapies or subsequent administrations of the proteasome inhibitors. Tumor vascularity is also a critical factor in how osteosarcoma patients respond to chemotherapy in the clinic. Patients with a higher micro-vessel density in their primary tumors had an improved response to treatment compared to those with reduced micro-vessel density [69]. While our work revealed that proteasome inhibitors were only effective against pulmonary metastases, which were better vascularized than primary tumors, pulmonary osteosarcoma metastases in patients are similarly better vascularized than primary tumors [70], providing hope that our data may translate to a patient context. Vascular endothelial growth factor (VEGF) may be a useful biomarker to identify osteosarcoma patients whose tumors may be most likely to respond proteasome inhibitors, given that serum levels of VEGF in osteosarcoma patients correlate well with intra-tumoral levels [71].

One approach to improving the bioavailability of the proteasome inhibitors in primary osteosarcomas may be to package them in liposomes that preferentially permeate through tumor vasculature, which has already proven to be effective against other models of solid tumors [28]. Alternatively, complementing proteasome inhibitors with therapeutic agents that can remodel tumor vasculature to improve drug uptake into tumor tissue may be a more viable approach. Recently published work [72] revealed that thalidomide can improve the uptake of cisplatin in solid tumors by promoting vascular maturity while simultaneously reducing the frequency of metastases by decreasing tumor angiogenesis. It would be interesting to see if thalidomide is able to enhance the efficacy of proteasome inhibitors as well. 

As this study was conducted with nude mice, which possess an innate immune system but lack T cells [73], the efficacy of both proteasome inhibitors may have been underestimated in our experiments. Other studies have demonstrated in pre-clinical models of solid tumors that bortezomib could sustain T-cell activation signals, synergistically enhancing survival of mice administered with naïve CD8+T-cells and sensitizing tumor cells to T-cell mediated lysis [74,75]. Future work should also investigate whether ixazomib has improved anti-osteosarcoma activity in immunocompetent mice.

## 4. Materials and Methods

### 4.1. Cell Lines

Parental human osteosarcoma cells KRIB, 143B and KHOS provided by Nicholas Saunders and derivatives expressing luciferase and mCherry [39] were cultured in DMEM media (Invitrogen; Waltham, MA, USA) supplemented with 10% FBS. Cell lines were authenticated by short tandem repeat profiling. All cell lines were cultured in humidified incubators at 37 °C in 5% CO_2_. For ex vivo treatment of human osteosarcoma cells, tumors were resected from mice and digested in 0.25% trypsin-EDTA (Thermo fisher scientific; Waltham, MA, USA) for 1 h at 37 °C and cultured in the conditions described above. For ex vivo sensitivity assays, cells were isolated from canine osteosarcomas harvested from dogs that were undergoing routine amputation of the affected limb as part of routine clinical care and not for the purposes of this study. Following amputation, 8-gauge core biopsies were taken from primary tumors, ground with a mortar and pestle in 1 mg/mL collagenase/dispase (Sigma-Aldrich; Castle Hill, NSW, Australia) and incubated with shaking at 800 rpm for 1 to 5 h at 37 °C. Cells were passed through a 0.4 µm cell strainer into α-MEM media (Lonza; Mount Waverley, VIC, Australia) supplemented with 10% FBS and 2 mM L-Glutamine, 100 U Penicillin and 0.1 mg/mL Streptomycin (Sigma-Aldrich; NSW, Australia).

### 4.2. Drugs

The drugs used in this study were bortezomib, ixazomib, carfilzomib (Selleck Chemicals; Houston, TX, USA), doxorubicin and cisplatin (Sigma; NSW, Australia).

### 4.3. Animal Studies

Animal experiments were conducted in accordance with Australian Code of Practice for the Care and Use of Animals for Scientific Purposes, as approved by the La Trobe Animal Ethics Committee (approval AEC 17–76).

Five to six-week-old BALB/c-Foxn1^nu/Arc^ (“nude”) mice were purchased from the ARC (Australia) and housed at La Trobe Animal Research facility in individual ventilated cages, with 12-h light/dark cycling and unrestricted access to food and water. Mice were monitored and weighed each day. Euthanasia was performed by CO_2_ asphyxiation or cervical dislocation. KRIB-luc intramuscular primary tumor models were generated and imaged as previously described [30]. Mice were ranked based on the bioluminescent value of their tumors one week after implantation of cells and alternately distributed. Mice bearing 143B-luc and KRIB-luc pulmonary metastases were generated and imaged as previously described [41]. Mice were imaged twice weekly until a lung tumor was detected, alternately allocated to treatment groups, and then treatment with drugs or saline commenced the following day. Mice were imaged once per week post tumor detection until the endpoint of the experiment.

Metastases were imaged by bioluminescence ex vivo at the endpoint of experiments. Mice were intraperitoneally injected with 150 mg/kg D-luciferin (Pure Science; Porirua, New Zealand) and humanely euthanized ten minutes later. Organs were removed from mice, placed in a small petri dish containing 15 mg/mL D-luciferin and imaged immediately. Circular regions of interest were drawn around organs to measure bioluminescence intensity using living image software version 4.7.2 (Perkin Elmer; Waltham, MA, USA) corresponding to the level of luciferase expressing tumor cells present.

Control mice were injected with saline intraperitoneally using a 27-gauge needle twice weekly for four weeks. Bortezomib was dissolved in PBS to 0.1 mg/mL and administered intraperitoneally with a 27-gauge needle twice per week for 4 weeks at 1 mg/kg. Ixazomib was dissolved in 10% (2-Hydroxypropyl)-β-cyclodextrin to 1 mg/mL and administered by oral gavage twice per week for 4 weeks.

### 4.4. Cell Viability Assays

Five hundred canine or 2000 human osteosarcoma cells respectively were seeded in white 96-well plates containing drugs or media to a final volume of 100 µL and incubated for a specified time. Luminescence, reflecting ATP levels in viable cells was measured using the CellTiter-Glo 2.0 kit (Promega; Fitchburg, WI, USA) as described [76].

### 4.5. Immunofluorescence

Tissue was incubated in formalin for at least 48 h before being embedded in paraffin wax. Four-micron sections were cut from paraffin blocks and mounted on Menzel-Glaser Superfrost Plus*^®^* microscope slides (Thermo fisher scientific; MA, USA). Slides were dried overnight at 37 °C before being incubated at 65 °C for 10 min. Slides were dewaxed, stained and cleared by two 4-min incubations in xylene (Amber scientific; Midvale, WA, Australia), two 2 min incubations in 100% ethanol, tap water for 5 min. Antigen retrieval was performed in a pressure cooker in 10 mM sodium citrate pH 6.0 at 110 °C for 10 min. Tissue sections were permeabilized with 1% Triton X-100 in PBS for 10 min at room temperature and blocked for one hour with PBS containing 1% BSA and 0.1% Tween20 for one hour at room temperature in a humidified chamber. Block solution was aspirated from the sections and stained with anti CD31-eFluor450 and isotype control rat IgG2 aκ-eFluor450 (1:200) (Thermo Fisher Scientific; Waltham, MA, USA) or anti CD44-FITC and isotype control rat IgG2a-FITC (1:300) (SouthernBiotech; Birmingham, AL, USA). All antibodies were diluted in block solution and incubated overnight at 4 °C in a humidified chamber. Sections were washed 3x in PBS containing 0.1% Tween20 for five minutes with shaking. Coverslips were mounted on slides with acqueous mounting medium (Abcam; Cambridge, UK). Slides were imaged on a Zeis 780 confocal microscope and images analyzed using Zen 3.1 blue edition. 

### 4.6. Pharmacodynamics

Tissue from treated and untreated mice was harvested and frozen immediately. Frozen tissue was mechanically homogenized on ice in hypotonic lysis buffer (50 mM HEPES, pH 8.0) [56]. Samples were sonicated using a microson ultrasonic cell disruptor (Misonix; Farmingdale, NY, USA), centrifuged at 13,000× *g* for 5 min at 4 °C and mixed 1:1 with stabilization solution (40 mM HEPES, 1 mM EDTA, 20% glycerol, pH 8.0). Protein was quantitated using a micro BCA kit (Thermo Fisher Scientific). LLVYase activity, which reflects chymotrypsin-like proteasome activity [56], was measured in reactions containing activity buffer (0.5 mM ATP, 1 mM DTT, 0.5 mg/mL BSA) and 100 μM Suc-LLVY-AMC (Enzo Life Sciences; Farmingdale, NY, USA). Fluorescence was measured using a Spectramax M5 (Molecular Devices; San Jose, CA, USA) and slope of the curve interpolated using GraphPad Prism 8.0 (GraphPad; San Diego, CA, USA).

### 4.7. Statistics

All statistical analyses were performed using GraphPad Prism 8.0 (GraphPad) software.

## 5. Conclusions

Ixazomib and bortezomib both inhibited the growth of KRIB-luc pulmonary osteosarcoma metastases but only ixazomib enhanced the survival of mice and inhibited the growth of aggressive pulmonary and abdominal 143B-luc osteosarcoma metastases. Neither proteasome inhibitor was effective against poorly vascularized primary tumors, implying that tumor vascularity is a determinant of efficacy of proteasome inhibitors as a class, against solid tumors. Our results warrant the further investigation of ixazomib as a potential new therapy for metastatic osteosarcoma, especially if future experiments demonstrate cooperation with currently used treatments in vivo. 

## Supplementary Materials

The following are available online at https://www.mdpi.com/2072-6694/12/5/1207/s1, Figure S1: Ixazomib toxicity.
