# Supplementary material for: The Proteasome Inhibitor Ixazomib Inhibits the Formation and Growth of Pulmonary and Abdominal Osteosarcoma Metastases in Mice
Source: Cancers (Basel). 2020 May 11;12(5):1207. doi: 10.3390/cancers12051207 (PMC7281181; doi:10.3390/cancers12051207)
Supplement: Supplementary file 1 [file cancers-12-01207-s001.pdf]

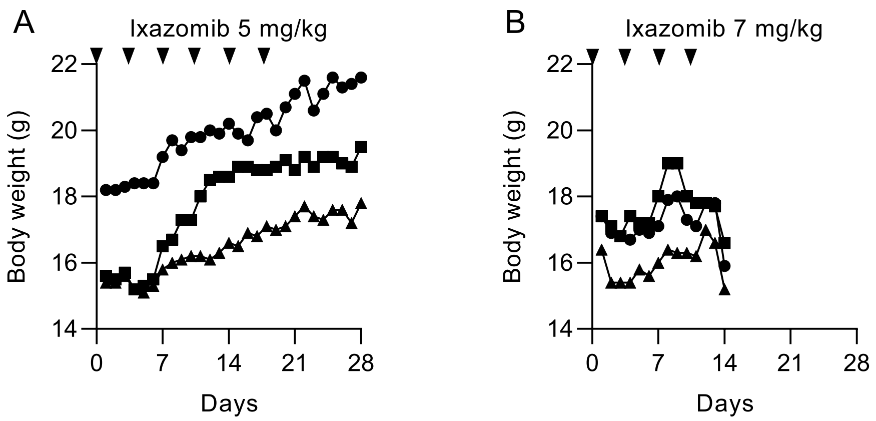

Supplementary Figure 1: BALB/c nude mice tolerate 5 mg/kg ixazomib administered twice weekly. Mice were administered with 5 mg/kg **(A)** or 7 mg/kg **(B)** ixazomib by oral gavage twice weekly and culled if acute weight loss exceeded 10%. Arrows indicate drug administration.
